# *Escherichiacoli* Nissle 1917 as a Novel Microrobot for Tumor-Targeted Imaging and Therapy

**DOI:** 10.3390/pharmaceutics13081226

**Published:** 2021-08-09

**Authors:** Qingyao Liu, Yongkang Gai, Yaqi Chen, Xiaoli Lan, Dawei Jiang

**Affiliations:** 1 Department of Nuclear Medicine, Union Hospital, Tongji Medical College, Huazhong University of Science and Technology, Wuhan 430022, China; lsqqy@hust.edu.cn (Q.L.); gykmail@hust.edu.cn (Y.G.); xiaoli_lan@hust.edu.cn (X.L.); 2 Hubei Key Laboratory of Molecular Imaging, Wuhan 430022, China; 3 Liyuan Hospital, Tongji Medical College, Huazhong University of Science and Technology, Wuhan 430077, China; cyaki31@hust.edu.cn

**Keywords:** *E. coli* Nissle 1917, microrobot, tumor colonization, bacteria-mediated tumor imaging, bacteria-mediated tumor therapy

## Abstract

Highly efficient drug delivery systems with excellent tumor selectivity and minimal toxicity to normal tissues remain challenging for tumor treatment. Although great effort has been made to prolong the blood circulation and improve the delivery efficiency to tumor sites, nanomedicines are rarely approved for clinical application. Bacteria have the inherent properties of homing to solid tumors, presenting themselves as promising drug delivery systems. *Escherichia coli* Nissle 1917 (EcN) is a commonly used probiotic in clinical practice. Its facultative anaerobic property drives it to selectively colonize in the hypoxic area of the tumor for survival and reproduction. EcN can be engineered as a bacteria-based microrobot for molecular imaging, drug delivery, and gene delivery. This review summarizes the progress in EcN-mediated tumor imaging and therapy and discusses the prospects and challenges for its clinical application. EcN provides a new idea as a delivery vehicle and will be a powerful weapon against cancer.

## 1. Introduction

At present, traditional chemotherapy shows unsatisfactory clinical efficacy due to the low tumor accumulation and severe side damage to normal tissues. Although conventional nanomedicine has made significant progress in improving tumor accumulation, most of the nanoparticles are captured by the reticuloendothelial system (RES), with only 0.7% (median) of the administered dose accumulated in the tumor site [1]. In addition, the poorly vascularized microenvironment and high interstitial fluid pressure of the tumor impede the arrival of nanoparticles to the hypoxic regions, leading to a significantly reduced antitumor effect [2,3]. Exploring new drug delivery systems with improved tumor targeting efficiency, deepened tumor penetration, and decreased tissue toxicities are urgently demanded.

**Figure 1:**
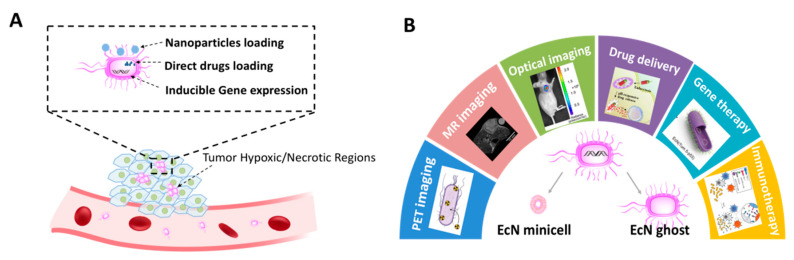
EcN-mediated tumor imaging and therapy. (**A**) Schematic illustration of the ability of preferential tumor colonization in hypoxic regions. EcN can be designed to load drugs or integrate nanoparticles and express exogenous genes; (**B**) Schematic diagram of the strategies of various imaging modalities and treatment patterns for EcN, EcN minicell, and EcN ghost.

More than 100 years ago, bacteria were identified in human tumors [4,5]. Since William B. Coley first used bacteria as an anticancer agent to treat malignant tumors, it has opened up a new field of bacteria-mediated tumor treatment. Nowadays, various bacterial species, such as Escherichia [6,7], Clostridium [8,9], Salmonella [10,11], and Bifidobacterium [12,13], have been found to selectively colonize and preferentially replicate within solid tumors, piquing increasing interest for tumor-targeted diagnosis and therapy. At present, the mechanism of tumor-targeting bacteria remains unclear. However, the following interactions have been identified to contribute to bacteria’s tumor-homing [14,15]: the hypoxic area of solid tumors provides the necessary survival environment for obligate anaerobes and facultative anaerobes, and the necrotic region provides enough nutrients needed for growth. In addition, the leaky vasculatures of the tumor facilitate the entering of bacteria, while the immunosuppressive tumor microenvironment inhibits their clearance. Preferential tumor accumulation and continuous growth make the bacteria a promising delivery vehicle. Bacteria can load chemotherapeutic drugs directly or integrate drug-carrying vehicles to achieve enhanced antitumor efficacy. In addition, the genes of bacteria can be engineered to express exogenous therapeutic genes and reporter genes for tumor therapy and in vivo imaging (Figure 1A).

Although bacteria-mediated tumor treatment demonstrates great promise in animal models, many challenges remain before their clinical translation. The most significant hurdle is clinical safety and effectiveness. Many bacteria, such as *S. typhimurium* and Listeria monocytogenes, are human pathogens that often require the deletion of the virulence genes to minimize toxicity. Despite the attenuation procedure, a problem with attenuated living bacteria as anticancer agents is that toxicity at the dose required for effective therapy and reducing the dose results in poor clinical efficacy [16]. The same problem has occurred in a Phase Ⅰ clinical trial of attenuated *S. typhimurium* strain VNP20009 in cancer patients. The clinical results were disappointing, and none of the patients achieved tumor elimination as expected at the maximum tolerable dose of VNP20009, but the maximum tolerable dose is still much lower than the dose required for effective therapy [17]. It is of great importance to providing bacteria-mediated vehicles for clinical application with improved efficiency and enhanced safety.

More attention has been focused on the promising potential of nonvirulent bacteria for tumor therapy. One of the most intensely studied non-pathogenic strains is *Escherichia coli* Nissle 1917 (EcN), an intestinal probiotic isolated from a soldier who resisted a severe outbreak of diarrhea during World War I [18]. It has been applied to treat various dysfunctions and diseases of the intestinal tract for almost 100 years [19]. EcN does not produce any secretion toxins and exhibits good tolerance at all ages, even in full/pre-term babies [20], proving it to be a safe agent in humans. Compared with *S. typhimurium* and Listeria monocytogenes, EcN can be directly applied to the human body without any attenuation process, showing a better safety profile. Robust tumor colonization is a prerequisite for bacteria-mediated tumor treatment. One study compared different enterobacterial strains for their ability to colonize solid breast tumors [21]. In contrast to S. Typhimurium (namely, *S. typhimurium* 14,028 and *S. typhimurium* SL1344) and other *E. coli* (*E. coli* 4608–58, *E. coli* CFT073, and *E. coli* Top10), the number of EcN colonized in the tumor was the same, but the number of EcN in liver and spleen was significantly reduced to almost no infected. In other words, EcN exhibits better tumor specificity and does not cause any harm to normal tissues compared with other bacteria. Based on its proven clinical safety and high tumor-specific replication, we selected EcN for further study.

Here in this review, we highlight the recent progress of EcN as a promising and versatile platform for biomedical applications, where EcN is engineered to be a bacterium-based microrobot for cancer imaging and therapy (Figure 1B). We then discuss the opportunities and challenges regarding its potential translation, hoping that future efforts can be gathered to facilitate EcN’s clinical practice.

## 2. Characteristics of EcN

EcN is a facultative anaerobic organism that proliferates mainly in the interface between the necrotic and hypoxic regions of tumors [22] and exists in rich oxygen areas [23], expanding their potential applications for various tumor types. Moreover, the special serum-sensitive lipopolysaccharide on the membrane of EcN promotes quick clearance from normal organs [24]. Researchers have systematically studied the biodistribution and quantitative tumor colonization of EcN in vivo [21]. It was found that the tumor/liver ratio of EcN colonization after intravenous injection was at least 10,000:1 in 4T1 tumor-bearing BALB/c mice, giving EcN a massive edge over traditional nanomedicine in terms of tumor accumulation and overall biodistribution profile. Interestingly, the average number of EcN found in tumors was significantly higher than the injected dose due to the colonization and proliferation of EcN. The minimum bacterial dose for successful colonization was 20,000 CFU, at which the average number in the tumor reached 10^8^ CFU. As the injection dose increases, the number of bacteria colonized in the tumor increases, but the bacteria in the liver and spleen grow accordingly. Moreover, the route of EcN injection, such as intravenous (i.v.), intraperitoneal (i.p.), and intertumoral (i.t.) injection, did not influence the tumor targeting and tumor-to-organ ratios. Oral administration of EcN confirmed that the bacteria crossed the gastrointestinal tract and colonized hepatic metastases [25]. Therefore, preferential tumor colonization of EcN may allow for flexible administration choices to meet specific clinical needs.

EcN has multiple peritrichous flagella that may drive it forward as a bio-engine [26]. Therefore, EcN can be developed into a self-propelled microrobot to break through the biological or pathological barriers to deliver therapeutic payloads [27]. The whole-genome sequencing of EcN has been completed [28], and methods for genetic modification of genomes and transformation of plasmid have been established to engineer EcN for heterologous gene expression [29,30,31]. Therefore, the therapeutic payloads can be drugs, expressed proteins, antigens, and immunoregulatory factors. However, constitutive expression of therapeutic factors may cause undesirable adverse effects, such as hepatic and splenic injury, so it is necessary to control the heterologous gene expression temporally and quantitatively. Weiss’s group established an in vivo remote control (IVRC) system to deliberate the external control of gene expression [32]. Three inducible promoter systems enabled EcN to remotely control and precisely regulate the kinetics of gene expression, and the L-arabinose–ParaBAD system showed the highest induce efficiency [33]. After oral administration or intraperitoneal injection of inducer L-arabinose, the expression of reporter gene luciferase in EcN colonized tumor reached its maximum after 6 h and stopped when L-arabinose was removed. Therefore, the controllable expression of EcN may provide a highly flexible and suitable treatment for individualized therapy. Precise regulation of the ECN number to control its proliferation in the tumor and expression of therapeutic agents will be of great significance to achieve spatiotemporal and quantitative imaging and treatment response. However, the underlying mechanisms of tumor targeting, and colonization of bacteria are complex and remain unclear. The influential factors may include the bacterial species used, types of tumor treated, and the tumor microenvironment [34]. Therefore, regulating the expression of bacteria may be a more practical means.

## 3. EcN-Mediated Tumor Imaging

To investigate the biological behaviors of EcN in vivo, close monitoring of its physical distribution and metabolic fate is essential. Since the distribution of bacteria in vivo, including normal organs and tumors, is mostly heterogeneous, invasive tissue sampling (such as biopsy) proved suboptimal to obtain a comprehensive overview of bacteria location and proliferation in living organisms. Researchers have developed many non-invasive imaging techniques for the visualization and repetitive monitoring of bacteria.

### 3.1. Optical Imaging

Optical imaging is highly efficient and sensitive and can be used for real-time observation of bacteria distribution in living bodies. Several studies have described the imaging of EcN by expressing green fluorescent protein (GFP) [35], red fluorescent protein (RFP) [31], and luciferase [36,37]. Choy et al. constructed a vector containing the luxCDABE operon for bioluminescent labeling of Gram-negative bacteria, allowing for accurate real-time tracking of bacteria in the living body [38]. Based on this technology, EcN demonstrated its specific tumor-seeking ability after i.v. administration [39]. Their findings set the basis of EcN-based microrobot for tumor imaging and the subsequent cancer treatment using gene-modified EcNs. However, it is difficult to adopt optical imaging widely as it suffers from limited penetration depth in clinical practice for human bodies.

### 3.2. MRI Imaging

Compared with optical imaging and nuclear imaging, MRI has higher spatial resolution and can simultaneously obtain anatomical and physiological information without ionizing radiation. Therefore, MRI has been used to image several kinds of tumor-targeted bacteria. MRI can be used to detect Clostridium novyi-NT spores labeled with iron oxide nanoparticles [40,41]. Magnetotactic bacteria AMB-1 injected intravenously can accumulate in the tumor and significantly enhance the magnetic resonance signal [42]. Ferritin is a type of protein for iron storage that widely exists in microorganisms, plants, animals, and other species. The H-chain of ferritin presents ferroxidase activity, which can turn Fe^2+^ into Fe^3+^ to form a superparamagnetic iron oxide particle [43]. Thus, the T2 relaxation time in MRI was shortened, and the final MR imaging showed a low signal area. At least seven systems related to iron absorption in EcN [44] make it very competitive in iron uptake. In a study that evaluated the function of three ferritins from bacteria as MRI reporter genes, when compared with archetypal ferritin and the smaller Dps-type ferritin, bacterioferritin expressed by EcN showed the highest contrast change in tumor-bearing mice, suggesting the most promise as a reporter gene for MRI imaging [45].

### 3.3. Nuclear Imaging

**Figure 2:**
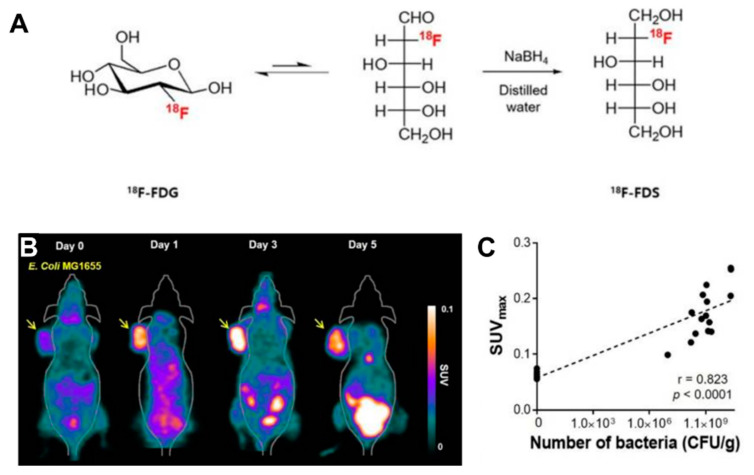
(**A**) [^18^F]-FDS PET imaging in CT26-bearing mice treated with *E. coli.* (**A**) PET imaging was performed at day 0, 1, 3, 5 after intravenous injection of *E. coli.* The radioactivity uptake of the tumor was significantly higher at day 1, 3, 5 than in pre-treatment. (**B**) Positive correlation between SUV_max_ and the number of viable bacteria. (**C**) Schematic illustration of the synthesis of [^18^F]-FDS from [^18^F]-FDG. Reproduced with permission from Jung-Joon Min, *Theranostics*; published by Ivyspring International Publisher, 2020.

Radionuclide-based molecular imaging, namely, PET and SPECT, is a powerful tool to assess physiological and pathological processes in vivo without penetration depth limitation. Currently, radiopharmaceuticals for bacterial imaging focus on tracking bacteria to differentiate sterile inflammation from infection [46]. Based on the tumor-specific colonization nature of EcN, radiopharmaceuticals monitoring EcN can be used for tumor imaging. The living body itself contains various background bacteria, so radiotracers must be highly specific to target injected EcN. The endogenous bacterial thymidine kinase gene (TK gene) of EcN has been shown to be an effective reporter gene for nuclear medicine imaging using radiolabeled pyrimidine nucleoside analogs, such as [^18^F]-FEAU, [^124^I]-FIAU, and [^125^I]-FIAU [39,47]. Since the substrate of bacterial TK presents poorly binding affinity with mammalian TK, the radiotracers mentioned above can selectively identify and locate bacteria in vivo [48]. PET Imaging with [^18^F]-FEAU exhibited high accumulation in tumors and a linear correlation with the number of colonized EcNs, offering precise information about the survival, proliferation, and number of the bacteria. A strategy of engineered EcN with exogenous reporter genes hSSTR2 has been reported for in vivo tumor visualization [49]. The outer membrane protein receptor FyuA of EcN can selectively recognize the ^64^Cu and ^89^Zr labeled metallophore yersiniabactin (YbT), which has a high affinity for transition metals [50]. A substantially higher PET signal was also observed in the EcN colonized tumor than that without the bacterial injection. PET tracers targeting bacteria-specific sugar metabolism have also been developed, and [^18^F]-FDS is the most representative one. [^18^F]-FDS, a synthetic analog of [^18^F]-FDG, has been shown to accumulate in Gram-negative Enterobacteriaceae selectively but not in mammalian or cancer cells. In PET imaging, the uptake of radioactivity in the tumor had a positive relationship with the number of viable bacteria, allowing a semiquantitative measure of bacterial density in the tumor [51] (Figure 2). The successful visualization and quantification of therapeutic *E. coli* by [^18^F]-FDS will make it possible to predict the therapeutic response, which could facilitate the clinical translation of bacteria-mediated tumor therapy.

Looking at the existing imaging technology, PET imaging or multimodal imaging with PET will be the most promising for the visualization of EcN in future human clinical trials due to its high sensitivity, unlimited penetration. Therefore, more efforts are needed to develop specific radiotracers that selectively target EcN rather than normal microbiotas or mammalian cells. Simple and faster synthetic approaches of radiolabeling for tracers are also required for clinical translation.

## 4. EcN-Mediated Tumor Therapy

The specific tumor targeting of EcN facilitates the establishment of a live platform for the delivery of cancer therapeutics. Three main strategies have been employed to achieve EcN-based tumor treatment: (1) to load the drug or nanoparticles as a microrobot for specific tumor delivery; (2) to engineer the EcN to express anticancer proteins for tumor management, and (3) to deliver the immuno-regulatory agent for cancer immunotherapy.

### 4.1. Direct Drug Delivery

For cancer treatment, the concentration of drugs in the tumor plays a key role in regulating the therapeutic effect. The EcN vehicles with self-propulsion ability may enhance the drug accumulation in tumor sites compared with passive drug diffusion. Once it reaches the tumor tissue, EcN drives itself to swim against the barriers of the tumor microenvironment and seek the hypoxic regions for colonization. Therefore, EcN-mediated drug delivery would penetrate the depth of the tumor to improve the antitumor efficacy. Doxorubicin (DOX) has been conjugated to EcN via acid-labile linkers, realizing a high DOX concentration in the tumor at the uptake value of ~12.9% of the injected dose per gram tissue (%ID/g) after 3 h of intravenous injection, which is much higher than the conventional nanocarriers [23].

**Figure 3:**
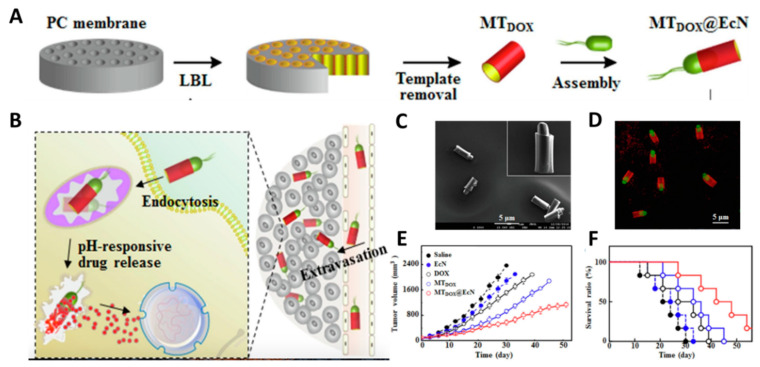
(**A**) Schematic illustration of the synthesis of MT_dox_@EcN; (**B**) Schematic illustration of the mechanism of MTDOX@EcN as a biorocket for drug delivery in tumor; (**C**) Typical SEM and (**D**) CLSM images of MTDOX@EcN. (**E**) Tumor inhibition and (**F**) survival rates of MT_dox_@EcN treatment in tumor-bearing mice. Reproduced with permission from Xiaohong Li, Chemical Engineering Journal; published by Elsevier, 2020.

However, nanomaterials have the advantages of multi-functionalization and modification. The integration of bacteria with nanomaterials offers a new combinational and synergistic therapeutic approach to reduce their respective limitations and achieve complementary advantages. An amphiphilic copolymer PM_TOS_/PM_DOX_ was obtained by conjugating poly(ethylene glycol) with doxorubicin (DOX) or α-tocopheryl succinate (TOS), and then immobilizing it onto the EcN through acid-labile linkers (namely EcN-PM_D/T_). In response to the acidic environment of the tumor, copolymers were released from EcN and self-assembled into hybrid micelles (M_D/T_) in situ. Then the GSH releases TOS and DOX to achieve tumor suppression. In treatment with free EcN, DOX/TOS mixtures, M_D/T_ hybrid micelles, and EcN-PM_D/T_, the EcN-PM_D/T_ conjugates exhibited enhanced tumor growth inhibition with a longer survival rate [52]. EcN can be successfully embedded into microtubes (MT_dox_@EcN) as a biorocket [53], which was confirmed by confocal laser scanning microscope (CLSM) images (Figure 3). The motion of EcN enhanced the extravasation of MT_dox_@EcN from the blood vessel, resulting in the high accumulation of DOX in the tumor. As shown in Figure 3E, the tumor inhibition ratio of MT_dox_@EcN (75.6%) is remarkably more prominent in vivo compared to EcN(20.6%), free DOX(36.1%), and MT_dox_(60.9%). Furthermore, MT_dox_@EcN exhibited a higher survival rate with a median survival time of 42 days, demonstrating stronger antitumor efficiency than other treatments. However, the microtubes also restricted the flagella movement, resulting in the lower velocity of MT_dox_@EcN (6.8 μm/s) than that of free EcN (9.8 μm/s) [23]. Reduction in motion ability directly influenced tumor targeting and penetration efficiency, which may be one reason MT_dox_@EcN did not eliminate tumor tissues in the antitumor experiment. The balance between the loading of nano cargos and the mobility of bacteria should be considered in the construction of the bacteria-nanomaterials system. In general, the integration of drug-carrying micro/nanomaterials with bacteria will enable them to work synergistically to achieve advanced antitumor effects.

To avoid the insecure factors caused by live bacteria and meet diverse medical needs, EcN can be prepared as minicells and bacterial ghosts (BGs). Minicells are the nanosized forms of bacteria that retain the same cytoplasmic components but lose the ability to proliferate due to genome deficiency [54]. BGs are the nonliving membranes shells of bacteria without cytoplasmic and DNA content [55]. Minicells and bacterial ghosts still retain the same tumor-targeting properties as their parent bacteria. The EcN-derived minicells displaying pHLIP could be used for delivering DOX and successfully invade the necrotic and hypoxic regions of orthotopic breast cancer [56]. EcN BGs were reported to be foreign antigen carriers and drug carriers for treating eye diseases [57].

### 4.2. Gene Therapy

EcN is a programmable vehicle designed to carry exogenous genes via Red/ET recombination or CRISP-Cas9 to express therapeutic proteins. EcN successfully expressed Tum-5, a powerful angiogenesis inhibitor. In mice bearing B16F10 mouse melanoma tumors, EcN (Tum-5) demonstrated remarkable tumor suppression after the upregulation of Tum-5 expression [58]. Then the Tum-5-p53 bifunctional proteins were constructed and engineered into EcN [59]. The decreased tumor volume and tumor weight indicated that the antitumor effect of EcN (Tum-5-p53) was significantly better than that of EcN (Tum-5) and EcN (p53) alone. Because of the powerful capacity of gene editing and packaging, EcN will play an important role in gene therapy.

### 4.3. Immunotherapy

**Figure 4:**
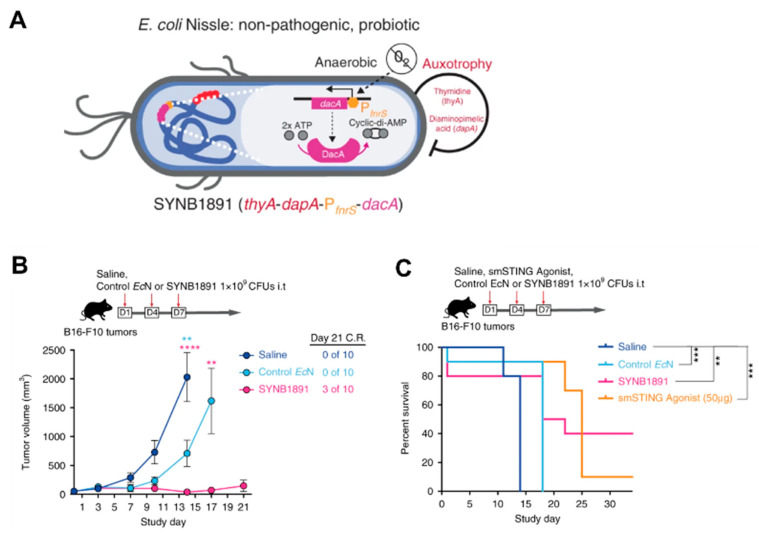
(**A**) Schematic illustration of the engineering EcN strain named SYNB1891; (**B**) Tumor inhibition and (**C**) survival rates of SYNB1891 treatment in B16F10 tumor-bearing mice. Reproduced with permission from Jose M. Lora, Nature Communications; published by Springer Nature, 2020. ** *p* = 0.0058 (blue stars), **** *p* < 0.0001 (pink stars), ** *p* = 0.0078 (pink stars), ** *p* = 0.006 (black stars), *** *p* = 0.0004–0.0006 (black stars).

Cancer immunotherapy aims to activate and harness the body’s immune system to attack malignant cells. However, more than 50% of patients failed to show a durable response to immunotherapy [60] but have immune-related adverse effects [61]. There is an urgent need for a targeted, localized, and sustained delivery of immunotherapeutic agents. Programmable EcN provides a convenient approach to meet the demand for immunotherapy. Gurbatri et al. [62] demonstrated PD-L1 and CTLA-4 antagonists expressed in EcN, enabling continuous and intratumoral checkpoint inhibitor production to induce a durable therapeutic response by a single injection. Due to its local delivery, the risk of systemic toxicities was greatly reduced. EcN has also been studied for its potential utility in vaccine development. Leventhal [63] designed an engineering EcN strain named SYNB1891 that expresses the STING-agonist cyclic di-AMP (CDA) to activate antigen-presenting cells (APCs) in the tumor. As shown in Figure 4, in B16F10 tumor-bearing mice with three i.t. injections over a week, SYNB1891 treatment resulted in complete tumor rejection compared to EcN alone. SYNB1891 treatment also exhibited greater long-term efficacy (40% survival) compared to treatment with the smSTING agonist (10% survival). Furthermore, the cured mice remained tumor-free after re-challenge for at least 60 days. It indicated that SYNB1891 treatment triggered efficacious antitumor immunity and immunological memory.

Localized administration of tumor-targeted EcN provides a distinctive option for tumor immunotherapy, inducing local immune responses to suppress tumor growth while avoiding systemic toxicity. Although the studies of live bacteria in tumor immunotherapy are still in their infancy, it opens up new opportunities and provides guidance for the development of future approaches to improve cancer immunotherapy.

## 5. The Challenges and Prospects

EcN emerges as a promising delivery platform for tumor-targeted imaging and therapy. Although significant progress has been made in preclinical studies, there are still challenges before extensive clinical translation. First, safety is the primary consideration for clinical use. Although EcN has been used as probiotics, the potential immunogenicity or autoimmune reactions must not be ignored, which may threaten immunocompromised patients with advanced-stage tumors. Recently, EcN has been reported to encode genes for colibactin, which may induce mutagenic DNA damage [64]. Deletion of certain virulence genes is a routine procedure to increase the safety of bacteria. It is noted that the attenuation process should be achieved without compromising the antitumor efficiency. After deleting the gene for colibactin, it is necessary to assess whether the ability of tumor-targeted colonization and self-replication has been affected. Another way to avoid the risk of colibactin is to use nonliving EcN in the form of bacterial ghosts, which do not contain DNA but retain tumor-targeting properties. Of course, more clinical observations are needed to assess the safety of long-term EcN use. Second, the genetic instability of exogenous genes introduced by gene transfer and plasmid mutation is another concern. Chromosome-plasmid balanced lethal system [65] or genome modification could improve the genetic stability. Third, live bacteria cannot be sterilized by conventional heating or filtration, bringing technical difficulty to clinical mass production. Thus, the whole production and purification process must be performed in dedicated clean rooms with good manufacturing practice (GMP) standards, following strict aseptic protocols for process monitoring.

**Figure 5:**
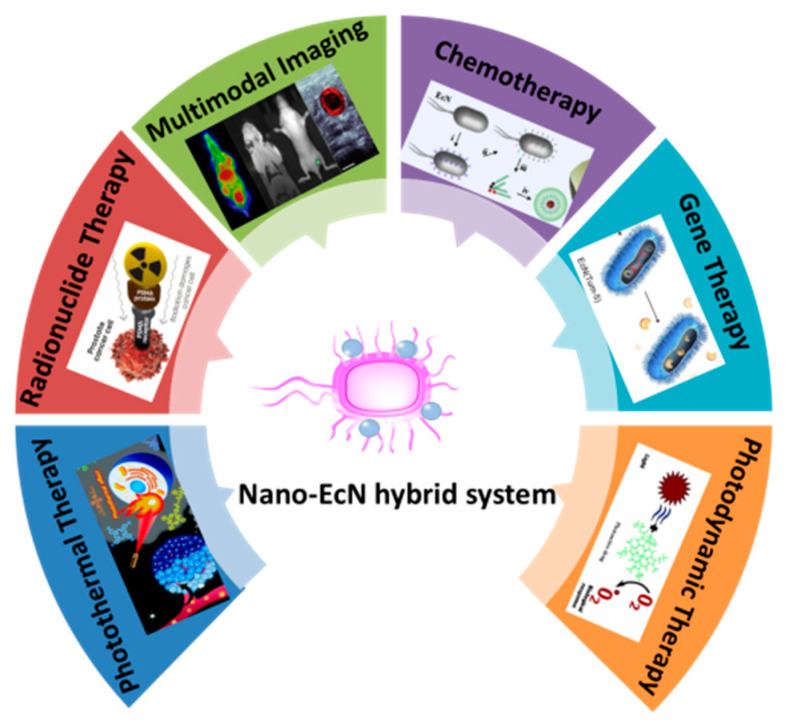
The future application of nano-bacteria hybrid system.

The research on EcN is still in its infancy, but it provides broad research space for researchers to expand its application in biomedicine. In the future, EcN may serve as a flexible platform to perform more complex tasks in a made-to-order fashion. Emerging imaging modalities, such as photoacoustic imaging [66], ultrasound imaging [67], and near-infrared (NIR) fluorescence imaging [68], could be installed in the EcN system for integrated imaging. Meanwhile, EcN-based therapeutic platforms are highly modular and may allow for convenient engineering of multiple payloads delivered as a microrobot for tumor therapy. In addition, we predict that the strategy of combining functional nanoparticles with EcN will be a possible direction for future research in this emerging field. In the past decades, the rapid development of nanomaterials has made a continuous effort to promote the progress of tumor treatment. The diversity of the nanomaterials endows EcN more functionality to achieve a variety of therapeutic paradigms, such as radionuclide therapy [69], photothermal therapy [70], and photodynamic therapy [71] (Figure 5). The combination therapy of EcN and nanomaterials will establish a nano-bacteria hybrid system that could reduce their respective limitations and exceed the advantages offered by each to maximize the therapeutic effect [72,73]. At the same time, further clinical trials are necessary to evaluate the safety, distribution, and metabolism of the nano-bacteria hybrid system. In summary, EcN shows excellent potential and brings new hope as an emerging treatment against tumors.

## Data Availability

This study did not report any data.
